# Sophisticated Adaptations of *Gregarina cuneata* (Apicomplexa) Feeding Stages for Epicellular Parasitism

**DOI:** 10.1371/journal.pone.0042606

**Published:** 2012-08-10

**Authors:** Andrea Valigurová

**Affiliations:** Department of Botany and Zoology, Faculty of Science, Masaryk University, Brno, Czech Republic; University of Melbourne, Australia

## Abstract

**Background:**

Gregarines represent a very diverse group of early emerging apicomplexans, parasitising numerous invertebrates and urochordates, and are considered of little practical significance. Recently, they have gained more attention since some analyses showed that cryptosporidia are more closely related to the gregarines than to coccidia.

**Methodology/Principal Findings:**

Using a combined microscopic approach, this study points out the spectacular strategy of *Gregarina cuneata* for attachment to host tissue and nutrient acquisition while parasitising the intestine of yellow mealworm larvae, and reveals the unusual dynamics of cellular interactions between the host epithelium and parasite feeding stages. Trophozoites of *G. cuneata* develop epicellularly, attached to the luminal side of the host epithelial cell by an epimerite exhibiting a high degree of morphological variability. The presence of contractile elements in the apical region of feeding stages indicates that trophozoite detachment from host tissue is an active process self-regulated by the parasite. A detailed discussion is provided on the possibility of reversible retraction and protraction of the eugregarine apical end, facilitating eventual reattachment to another host cell in better physiological conditions. The gamonts, found in contact with host tissue via a modified protomerite top, indicate further adaptation of parasite for nutrient acquisition via epicellular parasitism while keeping their host healthy. The presence of eugregarines in mealworm larvae even seems to increase the host growth rate and to reduce the death rate despite often heavy parasitisation.

**Conclusions/Significance:**

Improved knowledge about the formation of host-parasite interactions in deep-branching apicomplexans, including gregarines, would offer significant insights into the fascinating biology and evolutionary strategy of Apicomplexa. Gregarines exhibit an enormous diversity in cell architecture and dimensions, depending on their parasitic strategy and the surrounding environment. They seem to be a perfect example of a coevolution between a group of parasites and their hosts.

## Introduction

The alveolates (Alveolata), a major line of protists, include three extremely diverse groups of unicellular eukaryotes: ciliates, dinoflagellates and apicomplexans. Gregarines belong to the phylum Apicomplexa Levine, 1970, a large group characterised by the presence of a unique organelle called an apical complex, and which consists entirely of parasitic genera that infect a wide spectrum of invertebrates and vertebrates. Many of these are intensively studied etiologic agents of globally significant human disease, including malaria, toxoplasmosis and cryptosporidiosis. In contrast, gregarines are restricted to the internal organs and coelom of invertebrates and urochordates, and recently have been classified into three orders: Archigregarinorida Grassé, 1953; Eugregarinorida Léger, 1900; and Neogregarinorida Grassé, 1953 [1]. They are considered of no economic or medical significance and thus, despite their enormous diversity, the general biology of gregarines remains poorly understood. Recent phylogenetic analyses, however, have pointed out their close affinity with *Cryptosporidium*, and have drawn attention to this enigmatic group [2], [3].

Apicomplexans exhibit very specific adaptations for invading and surviving within their hosts, which have evolved under distinct evolutionary pressures, resulting in diverse attachment strategies and host-parasite interactions. In general, gregarines exhibit several known strategies for attachment to the host tissue: (i) intracellular or intratissular localisation with or without a reduced area of attachment in neogregarines; (ii) a mucron in archigregarines, monocystid eugregarines and some neogregarines; (iii) a simple epimerite in eugregarines and a few neogregarines; (iv) a complicated epimerite equipped with various structures, e.g. digitations, hooks or spines, hairs in eugregarines; (v) a sucker-like protomerite or modified protomerite with rhizoids in eugregarines. Eugregarines, similarly to cryptosporidia, are specific with their unique epicellular localisation [4], [5], [6], [7], [8]. Their sporozoites usually invade epithelial cells; however, some species are able to invade even the intercellular space. As the majority of eugregarines do not exhibit intracellular development, sporozoites generally develop into large extracellular vegetative stages, called trophozoites, exhibiting a high degree of cell polarity in that they possess an anterior part specialised for attachment to the host cell in general [9]. In intestinal species, the development of the trophozoite starts after sporozoite interaction with the microvillus border of the host epithelium, when apical organelles disappear and an epimeritic bud derived from the conoid forms at the apical end [4], [10]. The epimeritic bud gradually transforms into a specialised structure called the epimerite, which serves to anchor the parasite firmly to the host cell [4], [8], [10]. It is already known that epimerites of eugregarines parasitising herbivore hosts are usually simple button-shaped; however, they are much more complicated in carnivorous hosts, equipped with strong hooks, spines or numerous filaments [9].

The mechanism of nutrition acquisition in gregarines, however, is still poorly understood. Some authors attribute feeding function to the attachment organelles, such as the epimerite or mucron [9]. The higher concentration of host cell mitochondria and endoplasmic reticulum surrounding the epimerite, and the presence of mitochondria under the epimeritic cortical vesicle, indicate the existence of an active interaction between the gregarine epimerite and the host cell [11], [12]. Furthermore, the presence of organelles associated with nutritive function suggests that the epimerite is a metabolically active organelle [13], [14], [15]. Some eugregarine species are equipped with additional structures located in the grooves between the epicytic folds covering the gregarine body, resembling the micropores (diminished cell mouth) reported in other apicomplexans [11], [16], [17]. Thus, questions arise as to whether and under what circumstances the epimerite serves as a feeding organelle, and whether the micropore-like structures in gregarines are points implicated in pinocytosis [18] or are exclusively dedicated to mucus extrusion [19]. Similarly, the exact mechanisms responsible for trophozoite attachment to the host cell and for abandoning the host tissue at the end of development still remain enigmatic. There are two contradictory hypotheses on gregarine detachment from host tissue at the end of the trophozoite stage. One of them describes trophozoite detachment via epimerite retraction, self-regulated by the vegetative stage [8], while the other is based on gradual epimerite constriction facilitated by the supposed contractility of an osmiophilic ring surrounding the base of the epimerite and acting as a sphincter during the separation of the epimerite from the rest of the gregarine body [9], [10], [20], [21], [22]. All of these questions raised by conflicting data must be satisfactorily answered to clarify the parasitic strategies of gregarines and to better understand the evolutionary history of the phylum Apicomplexa.

This study endeavours to address the questions set out above and aims provide a new insight into the dynamics and architecture of the attachment site of *G. cuneata*. Unique relationships with the host epithelium, not only in trophozoites but also in more advanced developmental stages including gamonts, are described herein. Though there are few published works dealing with the life cycle and host specificity of *G. cuneata* [23], [24], [25], complete data on its early development and host-parasite interactions at the cellular level are still lacking. Based on personal observations of four eugregarine species (*Gregarina cuneata*, *G. polymorpha*, *G. steini* and *G. niphandrodes*) parasitising the yellow mealworm beetle *Tenebrio molitor*, in many aspects, *G. cuneata* appears to be the most spectacular of them all. Conclusions are supported by identification and detailed descriptions of structures involved in the formation of host-parasite interactions using a combined microscopic approach.

## Materials and Methods

Larvae of the yellow mealworm, *Tenebrio molitor* Linnaeus, 1758 (Coleoptera, Tenebrionidae) with eugregarine infection were obtained from colonies maintained in our laboratory. Gametocysts of *Gregarina cuneata* were collected from the faeces of infected larvae and placed in moist chambers at 25°C for maturation and dehiscence. Larvae sterilised of eugregarines were allowed to feed for 24 h on flour contaminated with the oocysts of *G. cuneata*, and were subsequently maintained on a sterile substrate. Insects were anesthetised with cold and dissected at different time points after feeding with eugregarine oocysts. Squash and/or wet smear preparations were investigated with the use of an Olympus BX51 light microscope.

For observations on living gregarines, different solutions, including phosphate buffered saline, Insect Ringer's solution or Minimum Essential Medium [3% bovine foetal serum with penicillin, streptomycin, amphotericin B and L-glutamine], were used to prepare squash preparations.

### Transmission electron microscopy

Parasitised intestines were fixed overnight at 4°C in freshly prepared 2.78% glutaraldehyde in 0.2 M phosphate buffer for transmission electron microscopy. The specimens were then washed for 1 h in phosphate buffer (pH 7.0), post-fixed in 1% osmium tetroxide in the same buffer for 3 h and dehydrated in an alcohol series, before embedding in Epon (Polybed 812). Sections were cut with glass knives and stained with uranyl acetate and lead citrate. Procedures for freeze etching follow Schrevel et al. [26] using the BAL-TEC BAF 060 freeze-etching system. Observations were made using a JEOL 1010 TEM.

### Scanning electron microscopy

Specimens were fixed overnight at 4°C in freshly prepared 3% glutaraldehyde in 0.2 M cacodylate buffer (pH 7.4), washed 3×15 min in cacodylate buffer, post-fixed in 2% osmium tetroxide in cacodylate buffer (pH 7.4) for 2 h at room temperature and finally washed 3×15 min in the same buffer. After dehydration in a graded series of acetone, specimens were critical point-dried using CO_2_, coated with gold and examined using a JEOL JSM-7401F field emission scanning microscope.

### Fluorescence microscopy

The *G. cuneata* cell suspension was washed in 0.2 M phosphate buffered saline (PBS), fixed for 15 min at room temperature in 4% paraformaldehyde in 0.2 M PBS, washed again, and permeabilised for 10 min in 0.1% Triton X-100 (Sigma-Aldrich). For direct fluorescence, samples were washed for 2 h in the antibody diluent (0.1% bovine serum albumin, 0.5% Triton X-100 and 0.1% sodium azide in 0.1 M PBS), incubated for 2 h at room temperature with fluorescein isothiocyanate (FITC)-phalloidin (Sigma-Aldrich) and then washed again in antibody diluent. Preparations were mounted in anti-fade mounting medium based on 2.5% DABCO (Sigma-Aldrich) mixed with glycerol and 0.1 M PBS. For indirect immunofluorescence, samples were incubated for 2 h at room temperature in rabbit anti-myosin antibody (smooth and skeletal, whole antiserum from Sigma-Aldrich; dilution 1∶5) or in mouse monoclonal IgG anti-actin antibody raised against *Dictyostelium* actin that recognises *Toxoplasma* and *Plasmodium* actin (provided by Prof. Dominique Soldati-Favre) diluted in PBS with 0.1% BSA (dilution 1∶500), washed three times in PBS for 10 min and incubated with FITC-conjugated anti-rabbit IgG (dilution 1∶40) or anti-mouse polyvalent immunoglobulins (1∶125) in PBS with 1% BSA at 37°C for 1 h. After washing in PBS, preparations were counterstained with Evans blue (1∶5000) and mounted. Controls were labelled with FITC-conjugated secondary antibody alone without the primary antibody. Preparations were observed and documented using an Olympus BX60 fluorescence microscope fitted with a WB filter cube, a fully motorized inverse epi-fluorescence microscope Olympus IX 81 equipped with Cell∧R imaging station or an Olympus IX80 microscope equipped with a laser-scanning FluoView 500 confocal unit (Olympus FluoView 4.3 software).

## Results

### Feeding stages of *Gregarina cuneata* under light microscopy

**Figure 1:**
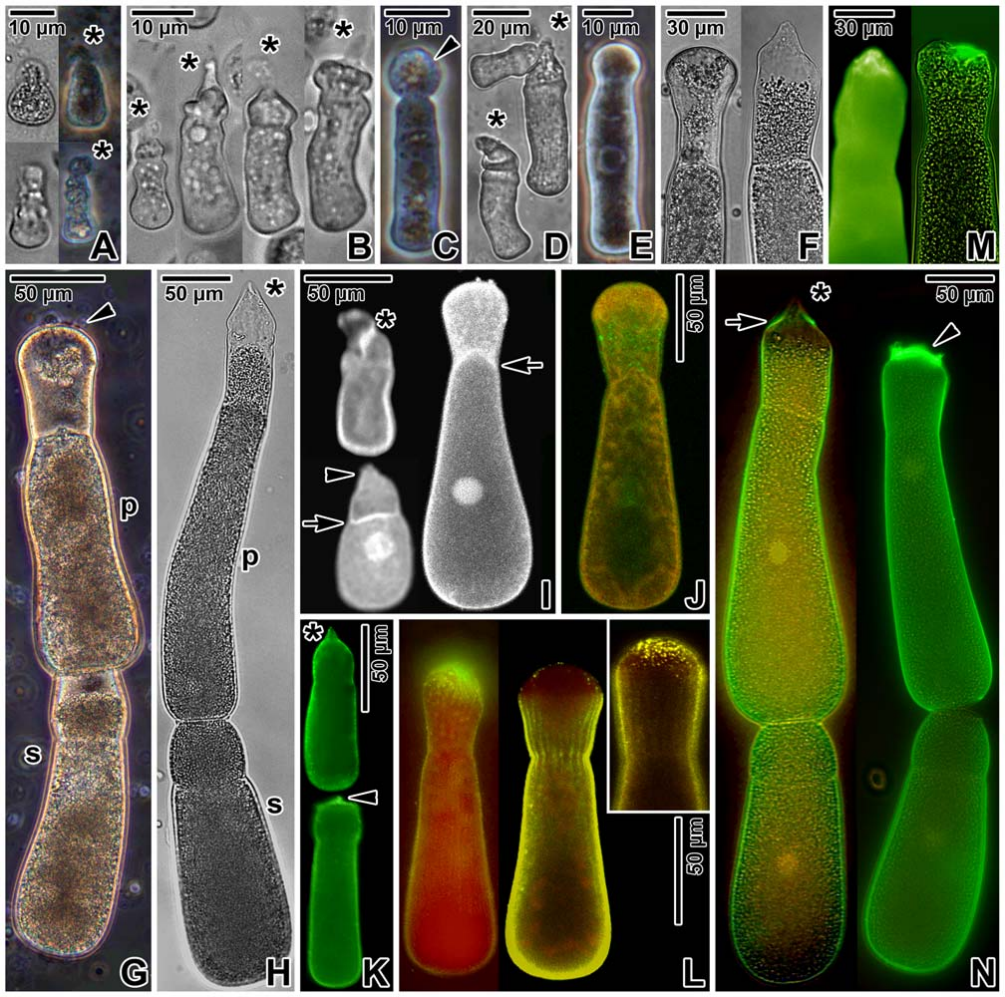
Early development of *Gregarina cuneata* observed using a light microscope. **A.** Earliest stages of trophozoites with developed epimerites (asterisks) under transmission light (left) and in phase contrast (right). **B.** Early trophozoites with polymorphous epimerites (asterisks). Transmission light. **C.** Detached maturing trophozoite with an epimerite surrounded by the host cell (arrowhead). Phase contrast. **D.** Three maturing trophozoites exhibiting obvious injury to their epimerites (asterisks) after forced separation from the host tissue by specimen processing. Transmission light. **E.** Maturing two-segmented individual exhibiting a well-developed protomerite and a cylindrical deutomerite. Note the rounded top of the protomerite lacking an epimerite. Phase contrast. **F.** Detailed view of the rounded protomerite top of a living gamont (left) and of the lance-shaped protomerite top of a chemically fixed gamont (right). Transmission light. **G.** Living gamonts associated in syzygy; primite (p), satellite (s). Note the rounded top of the primite protomerite with some remnants of the host tissue (arrowhead). Phase contrast. **H.** Chemically fixed gamonts associated in syzygy; lance-shaped top of the primite protomerite (asterisk), primite (p), satellite (s). **I.** Localisation of F-actin in early trophozoites (left) and maturing gamont (right); epimerite (asterisk), ruptured epimerite (arrowhead), septum (arrows) separating the protomerite from the deutomerite. Note that the septum (arrow) in the gamont is bulging into the protomerite. Direct fluorescence. **J.** Localisation of actin in maturing gamont. Note the patchy accumulation of actin with a very intense signal (green) in the protomerite cytoplasm. Immunofluorescence, counterstained with Evans blue. **K.** Localisation of myosin in trophozoites; epimerite (asterisk), ruptured epimerite (arrowhead). Immunofluorescence. **L.** Localisation of myosin in maturing individuals. The top of the protomerite exhibits more (left) or less (right) intense labelling, suggesting the presence of host tissue fragments. The *inset* shows the protomerite of more advanced stage of maturing gamont. Immunofluorescence, counterstained with Evans blue. **M.** Localisation of myosin in single maturing gamonts after detachment from host epithelium. The protracted (left) and retracted (right) protomerite tops exhibit strong labelling, suggesting the presence of host tissue fragments. Immunofluorescence; fluorescence and combination of fluorescence with transmission light. **N.** Localisation of myosin in mature gamonts associated in syzygies. Note the primite (left) with a lance-shaped top of the protomerite (asterisk) exhibiting distinct labelling in the peripheral area at its base (arrow) as well as the primite (right) with fragments of the host tissue covering its protomerite top (arrowhead). Immunofluorescence; combination of fluorescence with transmission light.

All vegetative stages of *Gregarina cuneata* exhibited epicellular development, i.e. sporozoites and trophozoites developed attached to microvillous sites of host epithelial cells; no developmental stage was observed penetrating under the host cell plasma membrane. When observed under the light microscope, it was difficult or even impossible to detect the earliest stages, such as sporozoites transforming into the trophozoites, and very young two-segmented trophozoites. These stages were small and inconspicuous (Figure 1A), and seemingly it was quite impossible to detach them from the host tissue without any damage. The only observed earliest trophozoite stages exhibited an irregular triangular shape, tapering towards their apical part with a polymorphous epimerite (Figure 1A - upper micrographs). The irregular shape of epimerites, as shown in these micrographs, seemed to be the consequence of mechanical damage due to the forced separation of the gregarine from the host epithelium during the processing of squash preparations. Three-segmented stages were irregularly shaped, with a cylindrical deutomerite widest at its posterior rounded end (Figure 1A - lower micrographs). Only few maturing detached trophozoites, with an apparently non-damaged epimerite still located on a relatively short protomerite and typical cylindrical deutomerite, were observed in squash preparations (Figure 1B). Their epimerites were usually conical to lance-shaped or prolonged of irregular shape. More often, however, were trophozoites released from the host epithelium and still bearing the affected host cell on their epimerites (Figure 1C) or trophozoites with ruptured epimerites (Figure 1D). The most frequently observed stages were small-sized hyaline gamont-like individuals (in size comparable to trophozoites) typical by a cylindrical protomerite, which was constricted at the septum and widely rounded at its apical top, and a prolonged cylindrical deutomerite widest at the posterior end (Figure 1E). These stages lacked an obvious epimerite. Gamonts of *G. cuneata* formed so-called early syzygies and thus only few non-associated gamonts could be found. This means that mature individuals transforming into gamonts (satellites) joined to individuals still attached to the host cell (future primites). Living (non-fixed) gamonts, either single or associated in syzygies, exhibited a prolonged cylindrical protomerite with a widely rounded top and cylindrical deutomerite (Figures 1F - left micrograph, 1G). The protomerite of paraformaldehyde fixed primites or single gamonts, however, showed a lance-shape hyaline apical top (Figures 1F - right micrograph, 1H). Light microscopic observations confirmed that the number of amylopectin granules increased with the age of the trophozoites and the cytoplasm of mature gamonts was fully packed with amylopectin, except in the region of the lance-shaped protomerite top in gamonts (Figures 1A–H).

### Localisation of actin and myosin

The homogenous distribution of the fluorescence signal throughout the surface of FITC-phalloidin labelled trophozoites and gamonts (Figure 1I) corresponded to the localisation of filamentous actin (F-actin) associated with the typical apicomplexan cell cortex. Labelling also confirmed the presence of F-actin in the peripheral region of growing epimerites. In all individuals, the fluorescence signal was more evident in the area of fibrillar septum separating the protomerite from the deutomerite. In addition, a large circular area in the deutomerite, suggestive of a nucleus, exhibited a more intense signal. In maturing single gamonts, the cytoplasm of protomerite exhibited more evident labelling of F-actin than that of deutomerite (Figure 1I), and it corresponded to the dot-like pattern of actin labelling restricted to the protomerite cytoplasm in individuals stained with the specific anti-actin antibody (proved to recognize the actin in *Toxoplasma* and *Plasmodium*) (Figure 1J). In comparison with the phalloidin-stained specimens, however, the gregarine cell cortex and septum exhibited only slight labelling of actin when stained with this antibody (Figure 1J).

Indirect immunofluorescence using rabbit anti-myosin antibody revealed the presence of myosin restricted to the epimerite region in trophozoites (Figure 1K) as well as to the gregarine cell cortex in trophozoites and gamonts (Figures 1K–L, 1N). In contrast to F-actin, no specific labelling of myosin corresponding to the septum was observed (Figures 1K–N). The intense fluorescence signal observed in the area of the obviously uneven protomerite top of some individuals lacking the epimerite most likely corresponded to a labelling of myosin in host tissue remnants covering the protomerite surface (Figures 1L–N). In addition, the intensely labelled apical end of the protomerite in some individuals seemed to be protracted or slightly retracted with an attached fragment of the host epithelium (Figure 1M). These data suggest that, in *G. cuneata*, more advanced stages than trophozoites remained in close contact with the host epithelium and detailed electron microscopic observations described below confirmed this assumption. In addition, some of the primites exhibited distinct circumscribed circular accumulation of myosin restricted to the periphery at the base of their lance-shaped protomerite top (Figure 1N). This structure might be related to the gamont feeding and/or attachment, however, its exact functions remains unclear as no comparable structure was observed under transmission electron microscope.

### Fine structure of feeding stages and their interactions with the host epithelium

**Figure 2:**
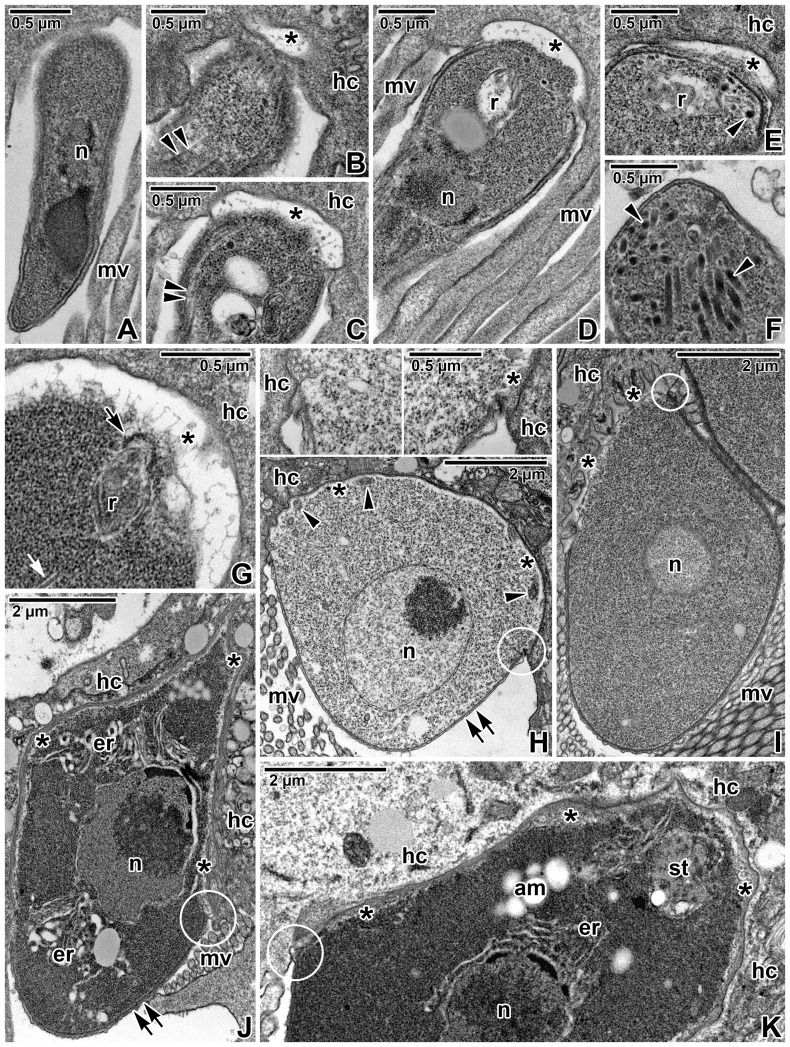
Early development of *Gregarina cuneata* observed using a transmission electron microscope. **A.** Invading sporozoite; host cell microvilli (mv), sporozoite nucleus (n). **B–G.** Sporozoite transforming into the trophozoite stage; conoid (arrow), developing epimeritic bud (asterisks), host cell (hc), host cell microvilli (mv), micronemes (arrowheads), microtubule (white arrow), nucleus (n), rhoptry-like organelle (r), subpellicular microtubules (double arrowheads). **H.** Early trophozoite stage. Note the anterior part of the gregarine, covered by a developing cortical vesicle (asterisks), causing an invagination of the host cell (hc) plasma membrane; host cell microvilli (mv), membrane fusion site (in circle), mitochondria (arrowheads), nucleus (n), pellicle (double arrow). Insets show details of the membrane fusion sites. **I.** Early trophozoite. Note the folded plasma membrane covering the cortical vesicle (asterisks) and forming numerous digitations; host cell (hc), host cell microvilli (mv), membrane fusion site (in circle), nucleus (n). **J.** Developing trophozoite; cortical vesicle (asterisks), endoplasmic reticulum (er), host cell (hc), host cell microvilli (mv), membrane fusion site (in circle), nucleus (n), pellicle with raising epicytic folds (double arrow). **K.** The apical end of another maturing trophozoite; amylopectin granules (am), cortical vesicle (asterisks), endoplasmic reticulum (er), host cell (hc), membrane fusion site (in circle), nucleus (n), unknown structure (st).

After entering the host intestine, invasive stages (sporozoites) excysted from the oocyst and invaded the host epithelium. During the invasion process, a slender sporozoite, tapering towards its posterior end, attached to the host cell plasma membrane via its apical part (Figure 2A) and, subsequently, the development of electron-lucent epimeritic bud started (Figures 2B–E, 2G). The apical cytoplasm of the invading parasite was packed with numerous electron-dense micronemes (Figures 2E–F) and a more or less translucent rhoptry-like organelle, passing through a conoid, that seemed to empty its contents at this stage (Figures 2D–E, 2G). In the course of transformation into a trophozoite, the sporozoite enlarged and attained a more round shape, and the epimeritic bud developed into an epimerite, gradually implanting into the host epithelial cell (Figures 2H–K).

The early trophozoite was attached to the host cell via an irregularly shaped epimerite, with its protodeutomerite hanging free into the intestinal lumen, and developed surrounded by host cell microvilli (Figures 2H–K). The irregularly shaped epimerite was still increasing in its size and was overlain by an indistinct cortical vesicle. The membrane-like structure, limiting the cortical vesicle on its cytoplasmic face, was discontinuous and often not apparent (Figures 2H–K). A few mitochondria were observed in the cytoplasm just beneath the epimeritic cortical vesicle (Figure 2H). The interface between the epimerite and the host cell was trilaminate, consisting of the epimerite and host plasma membranes with an intercellular space in between them. In some individuals, the epimerite plasma membrane covering the cortical vesicle formed numerous digitations and rhizoids (Figure 2I). The gradually expanding epimerite in the young trophozoite was very rich in endoplasmic reticulum connected to the nuclear envelope and often seen to be associated with the cortical vesicle covering the epimerite (Figures 2J–K). In addition, an apically located structure of unknown origin and function could be found close to (or in contact with) the cortical vesicle in some maturing trophozoites (Figure 2K). Considering its appearance and apical localisation, this structure could correspond to the residuum of the rhoptry-like organelle described above in the invading stages. The trophozoite was covered by a classical apicomplexan pellicle, consisting of a plasma membrane and an inner membrane complex, organised in raising longitudinal epicytic folds (Figures 2I–J). This three-layered pellicle, however, extended only to the protomerite top, at which point the inner membrane complex ceased and only the plasma membrane covered the embedded epimerite. The membrane fusion site between the epimerite plasma membrane, host cell plasma membrane and the membrane-like structure limiting the cortical vesicle was inconspicuous (Figures 2H–K).

**Figure 3:**
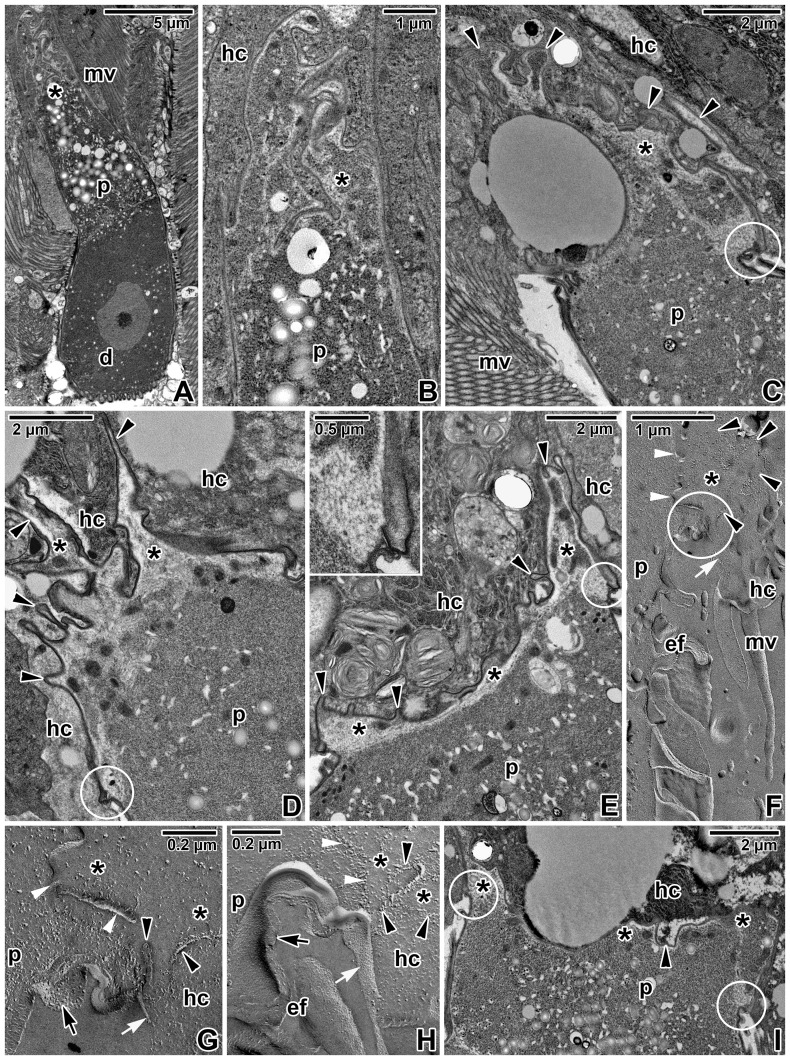
Trophozoites of *Gregarina cuneata* observed using a transmission electron microscope. **A.** Trophozoite with a well-developed epimerite (asterisk); deutomerite (d) with a nucleus, host cell microvilli (mv), protomerite (p). **B.** A more detailed view of the epimerite (asterisk) shown in Fig. 3A; host cell (hc), protomerite (p) cytoplasm packed with numerous inclusions. **C–E.** Decreasing epimerite (asterisks) forming numerous rhizoids and digitations (arrowheads) in more advanced stages of trophozoites as observed in different planes of sectioning; host cell (hc), host cell microvilli (mv), membrane fusion site (in circle), protomerite (p). The *inset* in Fig. 3E shows the membrane fusion site in detail. **F–H.** Host cell-epimerite interactions visualised by a freeze-etching technique. Fig. 3G shows a more detailed view of the membrane fusion site (in circle) from Fig. 3F. Note the border (arrowheads) between the epimerite and host cell (hc); cortical vesicle (asterisks), epicytic folds (ef) of the protomerite region (p), host cell microvilli (mv), host cell plasma membrane (white arrow), membrane-like structure limiting the cortical vesicle on its cytoplasmic face (white arrowheads), parasite plasma membrane (arrow). **I.** Trophozoite exhibiting a quite completely decreased epimerite (asterisks) with numerous mitochondria and gradual detachment (arrowhead) from host cell (hc), membrane fusion sites (in circles), protomerite (p).

**Figure 4:**
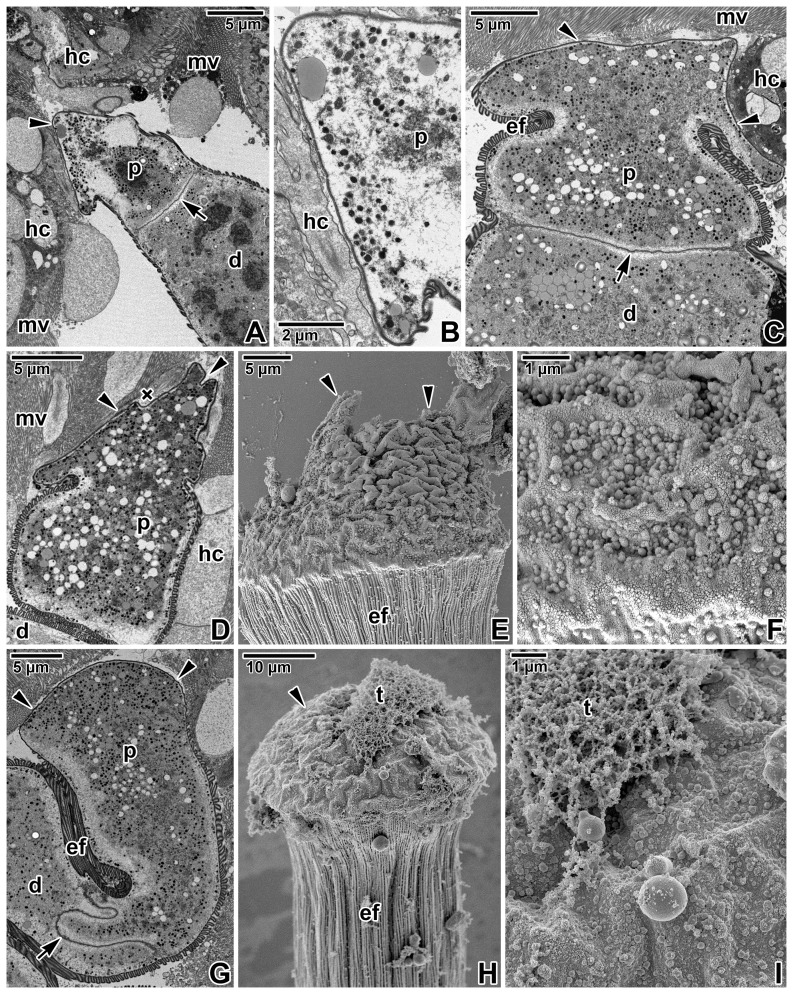
Gamonts of *Gregarina cuneata* observed using an electron microscope. **A.** Individual exhibiting a lance**-**shaped top (arrowhead) of the protomerite (p) in contact with a host cell (hc); deutomerite (d), microvilli (mv), septum (arrow). **B.** Higher magnification of the protomerite top shown in Fig. 4A. Note the close contact of protomerite (p) with the host cell (hc) in some areas. **C.** Longitudinal section of the protomerite (p) separated from the deutomerite (d) by a septum (arrow). Note that the tapered protomerite top, which is in contact (arrowheads) with host cell microvilli (mv), lacks the epicytic folds (ef) covering the rest of the gregarine body. **D.** A view of the protomerite (p) top (arrowheads) in close contact with the microvillous surface (mv) of host epithelial cells (hc); amorphous material (×), deutomerite (d). **E.** Scanning electron micrograph showing the protomerite top covered by a wrinkled plasma membrane; protomerite epicytic folds (ef). The apical end of the protomerite is obviously damaged (arrowheads), probably due to mechanical separation of the gregarine from the host tissue during specimen processing. **F.** A more detailed view of the protomerite top exhibiting small remnants of host tissue still attached to its plasma membrane. **G.** A general view of the protomerite (p) separated from the deutomerite (d) by a distinct septum (arrow); epicytic folds (ef). Arrowheads indicate the rounded protomerite top in contact with host cell microvilli. **H.** Scanning electron micrograph showing the rounded protomerite top (arrowhead) with a scrap of host tissue (t) attached; epicytic folds (ef) covering the rest of protomerite. **I.** A more detailed view of the plasma membrane covering the protomerite top shown in Fig. 4H; scrap of host tissue (t).

During trophozoite maturation, a thin septum developed and separated the protomerite from the deutomerite, retaining the large nucleus (Figure 3A). The epimerite appeared as an apical extension of the protomerite, growing through the host cell and interwoven with its plasma membrane, and overlain by an indistinct cortical vesicle (Figures 3A–B). The cytoplasm of the protomerite possessed numerous inclusions, including amylopectin granules. The cytoplasmic area interconnecting the epimerite and protomerite contained numerous membrane cisternae and vesicles (Figure 3B). In the course of trophozoite maturation, the epimerite seemed to decrease and the host cell and epimerite plasma membranes previously forming the trilaminate interface became indistinguishable from each other (Figures 3C–E). At this stage of gregarine epicellular development, the affected host cell exhibited some degree of vacuolation and in some sections cellular disorganisation (Figures 3C–E, 3I). The epimerite was irregularly embedded into the host cell (or in close contact with it), and formed numerous rhizoids or digitations of variable size and shape. The cytoplasm of these epimerite digitations appeared translucent, filled with numerous fine filamentous structures and mitochondria-like organelles underlying the cortical vesicle. The membrane fusion site, though not so prominent as in other eugregarines parasitising mealworms, was still visible (Figures 3C–E, 3I) and the freeze-etching technique revealed further details of its typical architecture (Figures 3F–H). According to the results of ultrathin sectioning, the border between the epimeritic cortical vesicle and the host cell was formed by the epimerite plasma membrane and the invaginated host plasma membrane, the second one of which was continuous with the plasma membrane covering surrounding microvilli. The parasite plasma membrane covering the epimerite was continuous with plasma membrane of the protomerite (Figure 3G). Similarly to the observations of ultrathin sections of mature trophozoites, epimerite and host cell plasma membranes were difficult to be distinguished from each other. The so-called ‘membrane-like structure’ limiting the cortical vesicle on its cytoplasmic face appeared as a membrane that was discontinuous in some areas, but often better visible than in ultrathin sections (Figures 3F–G). Nevertheless, it still remains unclear whether this membranous structure beneath the cortical vesicle was directly linked to the membrane fusion site or not (Figures 3G–H). Longitudinally oriented epicytic folds were a feature of both the trophozoite (Figure 3A) and gamont (e.g. Figure 4A) stages. In the course of trophozoite development, the decrease of epimerite proceeded and its detachment from host epithelium initiated. The protomerite top of individuals transforming from a trophozoite into a gamont exhibited an uneven surface with short rhizoid-like structures irregularly attached to the host tissue (Figure 3I) and thus resembling a retracted epimerite. The contact with the intestinal epithelium, however, was partially discontinuous, at least when observed in ultrathin sections.

Older stages, considered to be single maturing gamonts or primites, exhibited protomerites with broad lance-shaped apical ends (Figures 4A–D), similar to the light microscopic observations on chemically fixed parasites (Figures 1F, 1H, 1N), and usually in contact with host tissue. Less often, the protomerite top, contacting host microvilli, appeared widely rounded (Figures 4G–H). The apical end of the protomerite, regardless of its shape, was covered by a trilaminate pellicle lacking the typical organisation into longitudinal epicytic folds (Figures 4A–I). Under the scanning electron microscope, the cylindrical protomerite reached its maximum width at the interface between the apical part covered by a smooth pellicle and the posterior part with a pellicle organised into longitudinal epicytic folds (Figure 4H). The outer surface of the widely rounded protomerite top was wrinkled, bearing numerous non-specified globules of different size (Figures 4E–F). The localization and size of these globules corresponded to the myosin labelling of host tissue remnants still attached to the protomerite surface (as shown in Figure 1L). Many of the gamonts processed for scanning electron microscopy exhibited serious injury on the apical part of the protomerite (Figure 4E), often bearing scraps of host tissue (Figures 4H–I).

**Figure 5:**
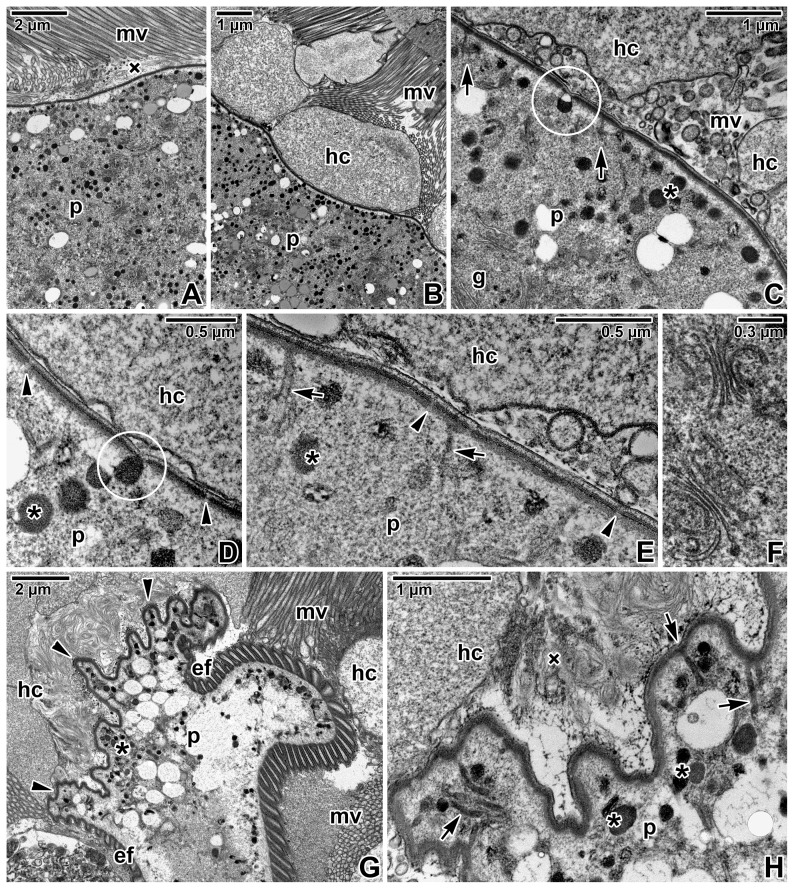
Host-parasite interactions in *Gregarina cuneata* as observed by transmission electron microscopy. **A, B.** A more detailed view of the protomerite top (p) in close contact with host microvilli (mv) or epithelial cells (hc); amorphous material (×). **C–E.** A detail of the protomerite (p) apical region covered by a three-layered pellicle underlined by a dense layer; Golgi apparatus (g), host cells (hc), microvilli (mv). Note the ducts (arrows) passing to the exterior, numerous dense bodies (asterisks), semi-empty (Fig. 5C) and filled (Fig. 5D) dense structures (in circle) directly linked to the pore-like structures (arrowheads) interrupting the inner membrane complex. **F.** Higher magnification of the Golgi apparatus frequently observed in the protomerite cytoplasm. **G.** A view of the protomerite top (p) exhibiting a more undulating pattern (arrowheads) in the area adjacent to the host epithelium (hc) with microvilli (mv); epicytic folds (ef), numerous dense bodies (asterisks). **H.** A higher magnification showing the protomerite (p) apical region with unusual duct-like structures (arrows). This region is obviously covered by a typical three-layered pellicle consisting of a plasma membrane and inner membrane complex underlined by a dense layer, but it lacks epicytic folds. Note that the inner membrane complex is discontinuous in a periodic pattern (interrupted by pore-like structures); amorphous material (×), dense bodies (asterisks), host cells (hc).

Detailed ultrastructural analysis of protomerite top found in close contact with host tissue revealed its unusual organisation, most likely dedicated to parasite food intake (Figures 5A–H). The contact of the gamont protomerite with host tissue was uneven, lacking any continuous intimate connection between the host cell and parasite plasma membranes (Figures 5B–E); more often, the protomerite top touched the host microvilli (Figure 5A). The apical end of the protomerite was covered by a smooth trilaminate pellicle, not organised in epicytic folds, with irregularly distributed pore-like structures (Figures 5C–E, 5H). In some sections, the protomerite top even showed a more undulated pattern (Figure 5G). Using higher magnification, a dense layer with non-membranous character, resembling the internal lamina usually underlining eugregarine epicytic folds, could be seen underlying the inner membrane complex at its cytoplasmic face (Figures 5D–E, 5H). The pore-like structures interrupting the inner membrane complex were more concentrated in some areas. In addition, structures similar to dense bodies, in some sections already half-emptied, could be seen in connection with them (Figures 5C–D). Unusual duct-like structures of unknown function could be found in the protomerite apical cytoplasm; usually, they were linked to the dense layer and in some sections their connection to abovementioned pore-like structures could be seen (Figure 5C). When observed under higher magnification, these structures appeared as elongated dense sacs passing through the inner membrane complex and plasma membrane and opening outwards (Figures 5E, 5H). The protomerite cytoplasm was packed with dense bodies, various vesicles and an abundant Golgi apparatus (Figure 5F).

Although the ultrastructural analysis revealed localized, mild pathological changes of the parasitised epithelium (usually limited to the affected cell), they seemed to be of minimal or no clinical significance. Despite often heavy infestation by *G. cuneata* in the host mid-gut, the experimentally infected larvae exhibited no obvious signs of sickness that could be considered to correlate with the progress of parasitisation. Parasitised larvae did not show any behavioural changes, weight loss or decreased food intake. In fact, the presence of eugregarines (regardless of eugregarine species) in experimentally as well as naturally infected mealworm larvae even seemed to increase the host growth rate and to reduce the death rate, and these larvae appeared to be more aggressive and agile in comparison to the gregarine-free individuals.

## Discussion

The observations on early development of *Gregarina cuneata* generally support previously published data on another eugregarines [4], [6], [7], [8]. Although *G. cuneata* trophozoites possess epimerite that slightly differs from those reported in other eugregarines parasitising mealworms, they also develop epicellularly and exhibit the same stages during their life cycle. Nevertheless, the later developmental stages exhibit more advanced adaptations to the epicellular parasitism and to the nutrient acquisition in intestinal environment, and details of these will be discussed below.

### Eugregarine attachment to host tissue

It has been determined that mesenteric epithelial cells are short-lived, living only four days in *T. molitor* [27]. The destiny of trophozoites with their epimerites still embedded in degenerating epithelial cells, often observed in insect hosts, is still unknown. Harry [28] described trophozoite detachment from the host tissue as a random and passive process at any stage of its development, depending on the degeneration of epithelial cells and their extrusion under the pressure of replacement cells. He referred to detached trophozoites still possessing epimerites observed in histological sections. On the contrary, recently published data [8], [11] revealed that epimerite detachment is an active process, and thus trophozoites most likely detach and search for a new host cell in better physiological conditions. Insofar as the vegetative phase of the eugregarine life cycle usually lasts longer than four days [6], [29], trophozoites must be adapted either to keeping the host cell alive during their development or for eventual reattachment to another cell. Therefore, Lucarotti [11] speculated about hypothetical reattachment of *Leidyana* trophozoites to younger cells after abandoning senescing cells, facilitated by a retractable epimerite and eugregarine gliding motility. The presence of contractile elements in the area of the epimerite [20], [21] and protomerite top [30], [31] serves as a more convincing argument in favour of the structural dynamics of these cell regions and epimerite retraction theory [8], [11]. Similarly, actin-like filaments demonstrated in the mucron of lecudinid eugregarine are considered to facilitate its adhesion to the host cell [20], [32]. As the parasite's fixation to host tissue might be of a temporary nature, the contact between the host cell and parasite attachment organelle must be very loose. Studies on attachment strategies of several eugregarines [4], [6], [7], [8], [10], [24] support this hypothesis in that they showed that, in the course of early trophozoite development, the gradually enlarging epimerite causes a deep invagination of the host plasma membrane, thus allowing the parasite to anchor to the surface of the host cell and to develop in an epicellular position. The interface between the epimerite and the host cell consists of epimerite and host plasma membranes, and a dense layer of unknown nature and origin in between them [4], [6], [7], [8], [10], [33]. The contact between the epimerite and the host plasma membrane is reported to be of the membrane-to-membrane type; lecudinid eugregarines establish an intimate contact with the host cell without an interspecific cell junction [34]. In fact, numerous detached trophozoites retaining intact epimerites, commonly observed in native or fixed squash preparations, are the evidence that no real fusion develops between the epimerite and host plasma membranes along the trilaminar interface. It seems that, simultaneously with trophozoite maturation, the host and epimerite membranes start to lose adhesion to one another and the epimerite gradually detaches from the epithelium. I repeatedly observed attached mature trophozoites of various species, in which the epimerite membrane was already separated from the host plasma membrane [7].

The feeding stages of *G. cuneata* exhibit an even more spectacular adaptation to epicellular parasitism. The atypical epimerite of *G. cuneata* develops from the epimeritic bud in accordance with other eugregarines, and later in development forms numerous digitations, deeply invaginating the plasma membrane on the luminal side of the affected host cell. Despite this parasite's firm anchoring to the brush border of the host cell, especially in mature stages when the host and epimerite membranes become almost indistinguishable from each other, *G. cuneata* trophozoites are able to detach while retaining an intact epimerite. Nevertheless, in contrast to other gregarines from mealworms, detached trophozoites of *G. cuneata* more often were found to have ruptured epimerites. Although the trophozoite development is more or less identical in all eugregarines from *T. molitor*, the destiny of *G. cuneata* mature trophozoites significantly differs in that they form so-called early syzygies, often found to be still attached to the host tissue. Surprisingly, the attachment site of attached primites significantly differs from the epimerite in younger stages, despite their resemblance at the light microscopic level. The ‘real’ epimerite disappears (retracts) and the top of the protomerite with a more or less undulating pattern remains in contact with the epithelium. The attachment by means of a modified protomerite could be facilitated by an increased flexibility of this region, as suggested by the dot-like accumulation of actin in the protomerite of *G. cuneata* as well as *G. polymorpha* [30]. Comparable attachment strategy has been reported from actinocephalid eugregarines [35] and it could be expected that protomerites modified for attachment act as feeding organelles in eugregarines lacking an epimerite. Similarly to *G. cuneata*, gamonts of some actinocephalids lose their small globular epimerites and subsequently attach by a modified, sucker-like protomerite [35]. The space between the epicytic folds of the attached protomerite and the host epithelium is filled by the microvilli embedded in a dense material interpreted to be adhesive. The authors speculate that this material could be produced by small dense (exocytic) vesicles in the protomerite apical cytoplasm. The space between the host microvilli and the *G. cuneata* protomerite top was also filled with an amorphous ropey material of unknown origin, probably serving as an adhesive. These observations are supported by the frequent presence of host tissue remnants attached to the *G. cuneata* protomerite, which was confirmed by the fluorescence labelling of myosin and not reported in other gregarines from mealworms.

### Nutrient acquisition in eugregarines

Nutrition of gregarines has been the subject of extensive debate for decades. There is evidence that feeding mode in gregarines depends on the long-term environmental conditions forming their niche. Correlations between trophozoite characteristics and the environment occupied within the host are discussed elsewhere [36]. The earliest diverging apicomplexans, archigregarines parasitising marine invertebrates, have retained myzocytosis as their principal mode of feeding [36]. The extensive folding of the pellicle covering the surface of large trophozoites of marine eugregarines seems to optimise surface-mediated nutrition (pinocytosis via micropores), and thus could explain the loss of an apical complex and myzocytosis in eugregarines along with the development of a bulky attachment apparatus, such as an epimerite or mucron [37].

The feeding strategy might even differ between distant eugregarine taxa. For instance, the supposed lytic effect of lecudinids on host cells indicates the nutritional function of the mucron via extracellular secretion of enzymes and absorption of digested host tissue [9]. In general, the epimerite cytoplasm contains many organelles usually associated with nutritive function [14]. Ghazali et al. [20] concluded that epimerites do not have a direct sucker function because of the absence of actin in the *G. blaberae* epimerite. On the contrary, our data confirmed the presence of F-actin in the epimerite region of eugregarines from mealworms [8,this study]. As host cells affected by attachment of *Gregarina* spp. vegetative stages usually do not show obvious pathological changes, the cortical vesicle and epimerite vacuoles most likely absorb nutrients via a mechanism based on membrane permeability [9]. Numerous mitochondria underlying the cortical vesicle, regularly observed in various eugregarine species [7], [8], [10], could provide the energy necessary for this putative absorption mechanism. The abundant endoplasmic reticulum repeatedly observed in the area of the expanding epimerite in young trophozoites of *G. cuneata* indicates the activation of metabolic pathways, probably involved in the synthesis and secretion of proteins and membrane manufacturing. The significant reduction in size of *G. cuneata* cortical vesicle might be related to the convoluted character of the epimerite, significantly increasing its absorptive surface, as reported in *Didymophyes* [12]. Similarly, the trilaminar junction between the mucron of the monocystid eugregarine *Nematocystis* and the host epithelial cell forms extensive folds to increase the surface contact between their apposing cell membranes [33]. Using radioisotopes, the study demonstrated that metabolites pass directly from the host cell to the trophozoites by crossing the attachment site of *Nematocystis*.

In gamonts of *G. cuneata* with their modified protomerites contacting the host epithelium, the pore- and duct-like structures were associated with the pellicle covering the protomerite top. Although the function of these structures remains uncertain, they are most likely involved in gamont nutrition and/or attachment. The apical localisation of numerous dense bodies, various vesicles and abundant Golgi apparatus in the protomerite cytoplasm of *G. cuneata* gamonts similarly indicates the involvement of protomerite top in the feeding.

The basic mechanisms of nutrient acquisition in gregarines, however, are still to be resolved. Despite all these studies attributing the major nutritional role to the attachment organelles, another possibility must be sketched, especially when considering the existence of gregarines growing in the coelomic fluid without an attachment to the host tissue. In addition, eugregarines usually continue to grow after detaching from the host tissue [9]. There are often speculations on the functionality of the micropore-like structures that are often observed in the spaces between epicytic folds [11], [16], [17], [18]; nevertheless, more elaborate analyses are needed to determine their involvement in gregarine nutrition and/or movement.

### Pathogenicity to insect hosts

Eugregarines are probably the most frequently encountered protists in insects and probably the most innocuous. As a rule, they are considered to be non-pathogenic to their hosts [38]; however, the real impact of eugregarine infection on host fitness and viability is still poorly understood. Misinterpretation of regular cellular processes in host tissue might significantly contribute to the controversy surrounding the pathogenicity of eugregarines. In addition, gregarines usually parasitise digestive epithelia that are the first to undergo autolysis after dissection, and this could hinder the correct determination of pathological changes induced by gregarines and distinguishing them from the post-mortem autolytic changes to the tissue. Some authors attributed pathogenicity mostly to trophozoites, which theoretically might cause some degree of damage to host tissue depending on the size and shape of their embedded epimerites [39]. The robust epimerite of *Ancyrophora* equipped with rigid hooks, however, does not appear to induce drastic damage to the host cell [14]. In fact, although eugregarines infecting the intestinal epithelium might cause certain damage to affected cells, continual regeneration of these cells accounts for the apparent harmless effect of the parasite. Usually, even if the eugregarine trophozoites destroy individual cells, the overall damage to epithelial tissue is negligible and easily repaired. Nevertheless, some species appear to reduce the host's fitness by occluding its gut and thus preventing the passage of food [11]. In addition, heavy infestations of gregarines in the mesenteron can have a significant impact on the host's nutritional state. As microvilli are important structures for efficient absorption and excretion, their destruction might limit the host digestive process and lead to its malnutrition with consequent weakening or even death. Gregarines parasitising intestinal caeca are even much more pathogenic, as they may cause the hypertrophy of parasitised cells or even rupture the caecal wall, leading to a secondary bacterial infection [40].

The eugregarines from mealworms were previously considered to be parasitic because of their negative impact on the development of larvae grown on a suboptimal diet [41]. Eugregarines occurring in larval *T. molitor* from our colonies, however, not only do not appear to harm their host, but could actually be considered to be mutualistic from a certain point of view. My personal long-term observations on *T. molitor* confirmed that despite heavy infection completely filling the larval mid-gut, the presence of eugregarines seems to have a positive impact on host development, fitness and longevity. Identical observations were made on naturally infected mealworm larvae from our laboratory colonies, usually parasitised by multiple eugregarine species (*G. cuneata*, *G. polymorpha* and *G. steini*) simultaneously. Similarly, Sumner [42] considered gregarines from mealworms to be symbiotic, and necessary for the normal growth and longevity of the host. This author even suggested that gregarines probably secrete essential substances such as vitamins or enzymes essential for larval growth. This study confirms that, though the affected epithelium shows some changes, parasitisation by *G. cuneata* seems to have no negative impact on host health that is essential for the gregarine survival. Despite high densities of vegetative stages attached to the host intestinal tissue, there is no evidence of direct damage to neighbouring epithelial cells. Vacuolation and eventual subsequent death of individual affected epithelial cells represented the most marked changes that could be considered to be associated with gregarine infection.

### Morphological changes of *Gregarina cuneata* in different environmental conditions

In the course of development, the epimerite of *G. cuneata* undergoes dramatic changes and some of these have been shown to be reversible depending on actual environmental conditions. Various stimuli from the trophozoite environment, such as changes in the chemical composition of the dissection buffer/host tissue, pH or temperature, seem to induce significant morphological changes of the epimerite and the protomerite top. Significant differences in the protomerite shape, evident especially in the primites, were noticed in this study prior to and after chemical fixation with different cross-linking fixatives - paraformaldehyde and glutaraldehyde. Non-fixed living gamonts exhibited a rounded protomerite top; however, those fixed with a paraformaldehyde solution often exhibited a lance-shaped protomerite top. These individuals are assumed to have been mechanically detached from the host tissue during specimen processing and simultaneously chemically fixed, thus maintaining the real shape of the protomerite when in close contact with the epithelium. Corresponding stages fixed with glutaraldehyde, however, did not exhibit such an obvious extension and tapering of their apical ends, although the protomerite top of gamonts was often slightly raised and covered by host tissue fragments. Only individuals found in contact with the host tissue after fixation preserved the lance-shaped protomerite top. Formaldehyde-based solutions fix the tissue by cross-linking proteins; its effects are reversible by excess water and the benefits include good tissue penetration. As glutaraldehyde is a larger molecule, the weakness of this fixative includes a slower rate of diffusion across membranes, resulting in poor tissue penetration and the changes caused by fixation are irreversible [43]. As the fixatives are known to induce remarkable changes in cell shape, rapid fixation by paraformaldehyde is thought to be the source of differences in the protomerite morphology in this study. This unexpected outcome of different fixations revealed morphological adaptations of *G. cuneata* to epicellular parasitism that are not commonly observed in living specimens. The facts discussed herein suggest that this gregarine is able not only retract but even repeatedly protract its apical end (epimerite or protomerite top dedicated to attachment) depending on environmental conditions and the need to reattach to another part of the host tissue.

### Conclusions

Gregarines are important from an evolutionary perspective because of their suspected deep-branching position within the phylum Apicomplexa. Although some ancestral features found in gregarines have given them a reputation of being a ‘primitive’ lineage of the Apicomplexa, the majority of them exhibit unique and novel adaptations to their environment [37]. A wide variety of morphological and functional adaptations that can be found in all gregarine taxa, along with the fact that only few invertebrate groups escaped infection with gregarines, indicates that they must be regarded as very successful and highly specialized parasites. The fascinating biology of these apicomplexans is derived from the basic cellular organization of the so-called zoite, an infectious developmental stage devoted to the invasion of host tissue. The detachment of vegetative stage from host tissue and its eventual reattachment, self-regulated by the parasite, might represent a higher degree of gregarine adaptation to epicellular development in hosts exhibiting a rapid epithelial replacement (e.g. insects). The modified protomerite of *G. cuneata* gamonts, serving for attachment to the host tissue and parasite feeding, indicates further adaptation of eugregarines for nutrient acquisition in older developmental stages that were previously considered to be non-vegetative. Such modifications for epicellular parasitism do not seem to be primitive ancestral characteristics, but rather advanced features occurring in some eugregarines in the course of their coevolution with the host.

## References

[1] Perkins FO, Barta JR, Clopton RE, Peirce MA, Upton SJ (2000) Phylum Apicomplexa Levine, 1970. In: Lee JJ, Leedale GF, Bradbury P, editor. An Illustrated Guide to the Protozoa. Second Edition, Vol. 1, Society of Protozoologists, Lawrence, Kansas, USA, pp. 190–369.

[2] Carreno RA, Martin DS, Barta JR (1999) *Cryptosporidium* is more closely related to the gregarines than to coccidia as shown by phylogenetic analysis of apicomplexan parasites inferred using small-subunit ribosomal RNA gene sequences. Parasitology Research 85: 899–904. 10540950 10.1007/s004360050655

[3] Templeton TJ, Enomoto S, Chen WJ, Huang CG, Lancto CA, et al (2010) A genome-sequence survey for *Ascogregarina taiwanensis* supports evolutionary affiliation but metabolic diversity between a gregarine and *Cryptosporidium* . Molecular Biology and Evolution 27: 235–248. 19778951 10.1093/molbev/msp226 PMC2877549

[4] Valigurova A, Hofmannova L, Koudela B, Vavra J (2007) An ultrastructural comparison of the attachment sites between *Gregarina steini* and *Cryptosporidium muris* . Journal of Eukaryotic Microbiology 54: 495–510. 18070327 10.1111/j.1550-7408.2007.00291.x

[5] Valigurova A, Jirku M, Koudela B, Gelnar M, Modry D, et al (2008) Cryptosporidia: Epicellular parasites embraced by the host cell membrane. International Journal for Parasitology 38: 913–922. 18158154 10.1016/j.ijpara.2007.11.003

[6] Valigurova A, Koudela B (2005) Fine structure of trophozoites of the gregarine *Leidyana ephestiae* (Apicomplexa : Eugregarinida) parasitic in *Ephestia kuehniella* larvae (Lepidoptera). European Journal of Protistology 41: 209–218.

[7] Valigurova A, Koudela B (2008) Morphological analysis of the cellular interactions between the eugregarine *Gregarina garnhami* (Apicomplexa) and the epithelium of its host, the desert locust *Schistocerca gregaria* . European Journal of Protistology 44 197–207. 18304787 10.1016/j.ejop.2007.11.006

[8] Valigurova A, Michalkova V, Koudela B (2009) Eugregarine trophozoite detachment from the host epithelium via epimerite retraction: Fiction or fact? International Journal for Parasitology 39: 1235–1242. 19460380 10.1016/j.ijpara.2009.04.009

[9] Schrevel J, Philippe M (1993) The gregarines. In: Kreier JP, editor. Parasitic Protozoa Second edition, Vol. 4, Academic Press, pp. 133–245.

[10] Tronchin G, Schrevel J (1977) Chronology of ultrastructural changes during growth of *Gregarina blaberae* . Journal of Protozoology 24: 67–82. 405485 10.1111/j.1550-7408.1977.tb05282.x

[11] Lucarotti CJ (2000) Cytology of *Leidyana canadensis* (Apicomplexa : Eugregarinida) in *Lambdina fiscellaria fiscellaria* larvae (Lepidoptera : Geometridae). Journal of Invertebrate Pathology 75: 117–125. 10772324 10.1006/jipa.1999.4911

[12] Hildebrand HF (1976) Electron-microscopic investigation on evolution stages of trophozoite of *Didymophyes gigantea* (Sporozoa, Gregarinida). 1. Fine structure of protomerite and epimerite and relationship between host and parasite. Zeitschrift Fur Parasitenkunde-Parasitology Research 49: 193–215. 10.1007/BF00380590 824879

[13] Ormieres R (1977) *Pyxinia firmus* (Leger, 1892), eugregarine parasite of Coleoptera *Dermestes frischi* Kugel - Ultrastructural study. Zeitschrift fur Parasitenkunde-Parasitology Research 53: 13–22.

[14] Baudoin J (1969) Sur l'ultrastructure de la région antérieure de la grégarine A*ncyrophora puytoraci* . Protistologica 5: 431–439.

[15] Schrevel J, Vivier E (1966) Étude de l'ultrastructure et du role de la région antérieure (mucron et épimérite) de grégarines parasites d'annélides polychètes. Protistologica 2: 17–28.

[16] Talluri MV, Dallai R (1983) Freeze-fracture study of the gregarine trophozoite: II. Evidence of “rosette" organization on cytomembranes in relation with micropore structure. Bolletino di zoologia 50: 247–256.

[17] Dyakin AY, Simdyanov TG (2005) The cortical zone of skittle-like cells of *Urospora chiridotae*, a gregarine from an apode holothuria *Chiridota laevis* . Protistology 4: 97–105.

[18] Vivier E, Devauchelle G, Petitprez A, Porchet-Hennere E, Prensier G, et al (1970) Observations on comparative cytology in sporozoans. Part 1. The sperficial structures in vegetative forms. Protistologica 6: 127–150.

[19] Schrevel J (1972) Polysaccharides of cell-surface of gregarines (Protozoa Parasites). 1. Ultrastructure and cytochemistry. Journal of Microscopy-Oxford 15: 21–40.

[20] Ghazali M, Philippe M, Deguercy A, Gounon P, Gallo JM, et al (1989) Actin and spectrin-like (Mr = 260–240 000) proteins in gregarines. Biology of the Cell 67: 173–184.

[21] Ghazali M, Schrevel J (1993) Myosin-like protein (M(r)175,000) in *Gregarina blaberae* . Journal of Eukaryotic Microbiology 40: 345–354. 8508173 10.1111/j.1550-7408.1993.tb04927.x

[22] Tronchin G, Philippe M, Mocquard JP, Schrevel J (1986) Life cycle of *Gregarina blaberae* - Description, chronology, study of the growth, influence of the cycle of the host *Blaberus craniifer* . Protistologica 22: 127–142.

[23] Ruhnke TR, Janovy J (1990) Life history differences between 2 species of *Gregarina* in *Tenebrio molitor* larvae. Journal of Parasitology 76: 519–522.

[24] Devauchelle G (1968) Étude ultrastructurale du développement des grégarines du *Tenebrio molitor* L. Protistologica 4: 313–332.

[25] Ruhnke TR, Janovy J (1989) The site specificity of 2 species of *Gregarina* in *Tenebrio molitor* larvae. Journal of Protozoology 36: 428–430.

[26] Schrevel J, Caigneaux E, Gros D, Philippe M (1983) The 3 cortical membranes of the gregarines. 1. Ultrastructural organization of *Gregarina blaberae* . Journal of Cell Science 61: 151–174. 6411745 10.1242/jcs.61.1.151

[27] Thomas D, Gouranto J (1973) Durée de formation des cristaux protéiques intranucléaires de l'intestin moyen de *Tenebrio molitor* . Journal of Insect Physiology 19: 515–522.

[28] Harry OG (1965) Studies on early development of eugregarine *Gregarina garnhami* . Journal of Protozoology 12: 296–305. 4955389 10.1111/j.1550-7408.1965.tb01856.x

[29] Harry OG (1970) Gregarines: their effect on the growth of the desert locust (*Schistocerca gregaria*). Nature 225: 964–966. 16056839 10.1038/225964a0

[30] Heintzelman MB (2004) Actin and myosin in *Gregarina polymorpha* . Cell Motility and the Cytoskeleton 58: 83–95. 15083530 10.1002/cm.10178

[31] Heintzelman MB, Mateer MJ (2008) GpMyoF, a WD40 repeat-containing myosin associated with the myonemes of *Gregarina polymorph*a. Journal of Parasitology 94: 158–168. 18372636 10.1645/GE-1339.1

[32] Schrevel J, Vivier E (1966) Etude au microscope électronique de la région antérieure des Grégarines: le mucron de *Lecudina pellucida* (Koll.) Mingazzini et l'épimérite de *Sycia inopinata* Léger. Protistologica 2: 17–28.

[33] MacMillan WG (1973) Gregarine attachment organelles - structure and permeability of an interspecific cell junction. Parasitology 66: 207–214.

[34] Desportes I, Theodorides J (1986) *Cygnicollum lankesteri* n.sp., grégarine (Apicomplexa, Lecudinidae) parasite des annélides polychètes *Laetmonice hystrix* et *L. producta*; particularités cytologiques de l'appareil de fixation et implications taxonomiques. Protistologica 22: 47–60.

[35] Cook TJP, Janovy J, Clopton RE (2001) Epimerite-host epithelium relationships among eugregarines parasitizing the damselflies *Enallagma civile* and *Ischnura verticalis* . Journal of Parasitology 87: 988–996. 11695420 10.1645/0022-3395(2001)087[0988:EHERAE]2.0.CO;2

[36] Leander BS, Lloyd SAJ, Marshall W, Landers SC (2006) Phylogeny of marine gregarines (Apicomplexa) - *Pterospora*, *Lithopystis* and *Lankesteria* - and the origin(s) of coelomic parasitism. Protist 157: 45–60. 16352468 10.1016/j.protis.2005.10.002

[37] Leander BS (2008) Marine gregarines: evolutionary prelude to the apicomplexan radiation? Trends in Parasitology 24: 60–67. 18226585 10.1016/j.pt.2007.11.005

[38] Henry JE (1981) Natural and applied control of insects by protozoa. Annual Review of Entomology 26: 49–73.

[39] Lipa JJ (1967) Studies on gregarines (Gregarinomorpha) of arthropods in Poland. Acta Protozoologica 5: 97–179.

[40] Tanada Y, Kaya HK (1993) Insect pathology. Academic Press, San Diego, CA, USA.

[41] Harry OG (1967) The effect of a eugregarine *Gregarina polymorpha* (Hammerschmidt) on the mealworm larva of *Tenebrio molitor* (L.). Journal of Eukaryotic Microbiology 14: 539–547. 10.1111/j.1550-7408.1967.tb02039.x 4973771

[42] Sumner R (1932) Influence of gregarines on growth in the mealworm. Science 78: 1. 10.1126/science.78.2015.125 17749825

[43] Dykstra MJ (1993) A manual of applied techniques for biological electron microscopy. Plenum Press, New York, N.Y, USA.

